# A New Variant of the Capsule 3 Cluster Occurs in *Streptococcus pneumoniae* from Deceased Wild Chimpanzees

**DOI:** 10.1371/journal.pone.0025119

**Published:** 2011-09-28

**Authors:** Dalia Denapaite, Regine Hakenbeck

**Affiliations:** Department of Microbiology, University of Kaiserslautern, Kaiserslautern, Germany; University of Osnabrueck, Germany

## Abstract

The presence of new *Streptococcus pneumoniae* clones in dead wild chimpanzees from the Taï National Park, Côte d'Ivoire, with previous respiratory problems has been demonstrated recently by DNA sequence analysis from samples obtained from the deceased apes. In order to broadenour understanding on the relatedness of these pneumococcal clones to those from humans, the gene locus responsible for biosynthesis of the capsule polysaccharide (CPS) has now been characterized. DNA sequence analysis of PCR fragments identified a cluster named *cps*3_Taï_ containing the four genes typical for serotype 3 CPS, but lacking a 5′-region of ≥2 kb which is degenerated in other *cps*3 loci and not required for type 3 biosynthesis. CPS3 is composed of a simple disaccharide repeat unit comprising glucose and glucuronic acid (GlcUA). The two genes *ugd* responsible for GlcUA synthesis and *wchE* encoding the type 3 synthase are essential for CPS3 biosynthesis, whereas both, *galU* and the 3′-truncated gene *pgm* are not required due to the presence of homologues elsewhere in the genome. The DNA sequence of *cps*3_Taï_ diverged considerably from those of other *cps*3 loci. Also, the gene *pgm*_Taï_ represents a full length version with a nonsense mutation at codon 179. The two genes *ugd*_Taï_ and *wchE*_Taï_ including the promoter region were transformed into a nonencapsulated laboratory strain *S. pneumoniae* R6. Transformants which expressed type 3 capsule polysaccharide were readily obtained, documenting that the gene products are functional. In summary, the data indicate that *cps*3_Taï_ evolved independent from other *cps*3 loci, suggesting the presence of specialized serotype 3 *S. pneumoniae* clones endemic to the Taï National Park area.

## Introduction

*Streptococcus pneumoniae* is one of the major bacterial human pathogens. Its polysaccharide capsule is an essential virulence factor [1]–[6]. In fact, the capsule gene cluster appears to be among the few components of *S. pneumoniae* described as virulence factors that distinguishes the pathogen from its closest commensal relative *S. mitis* [7]. Up to now over 90 capsular serotypes have been described that can be distinguished immunologically by antisera specific for the capsule polysaccharide (CPS), biochemically and genetically [8]–[11]. All *cps* clusters are located at a specific region in the genome flanked by conserved sequences of the two genes *dexA* and *aliA* [10].

The capsular serotype is also an important epidemiological marker for *S. pneumoniae*. Clones of genetically closely related strains can be characterized by multi locus sequence typing (MLST), i.e. comparative sequence analysis of seven house keeping genes, and thus individual strains are characterized by their allelic profile which constitutes the sequence type (ST) [12]. Generally, isolates with the same ST share the same serotype, although serotype switch occurs occasionally due to horizontal gene transfer of capsular genes [13]–[16].

*S. pneumoniae* is considered to be a human specific pathogen. Nevertheless pneumococci have been isolated from a variety of animals held in captivity (pets, zoo or laboratory animals), either as carriage isolates or causing a variety of disease symptoms [17]–[23]. There is only one case where *S. pneumoniae* were demonstrated in wild animals [24]. DNA sequencing using samples obtained from deceased wild chimpanzees from the Taï National Park revealed genes encoding typical *S. pneumoniae* proteins such as the major autolysin LytA, pneumolysin Ply, and the penicillin binding protein 2× (PBP2×). Moreover, MLST analysis identified two new clones that have not been found within the human population including workers on the Taï chimpanzee project. The closest human isolates differed in four out of seven alleles, and it has been suggested that *S. pneumoniae* virulent to great apes occur endemically in this area [24].

Since live bacteria could not be isolated from the wild chimpanzees, we have used DNA samples from three apes covering both STs to investigate the capsular type of the *S. pneumoniae* clones. Recently, a multiplex PCR scheme has been developed to differentiate 29 serotypes most common in the US [25]. In the present study a modulated system was used which covers the serotype distribution in Africa (http://www.cdc.gov/ncidod/biotech/strep/pcr.htm). The results document the presence of genes involved in CPS of type 3 in all samples. Comparison with known sequences of the *cps*3 locus from human isolates revealed major differences. Transformation experiments were performed using the laboratory strain R6 as recipient to verify their function.

## Results

### PCR amplification of the cps cluster from chimpanzee samples

In order to identify genes related to biosynthesis of the pneumococcal capsule, first a multiplex PCR was applied on DNA samples obtained from three chimpanzees (here referred to as ‘Taï’ samples) representing the three ape communities and the two *S. pneumoniae* clones identified by MLST analysis previously [24]. Each of the seven PCR reactions includes four to five primer pairs specific for distinct *cps* clusters. In addition, each reaction contains one primer pair which is specific for the gene *cpsA* (*wzg*) which is present in all *cps* clusters and thus serves as positive control ([25]. Forty serotype specificities are covered by a modulated version to include clinical specimen from Africa (http://www.cdc.gov/ncidod/biotech/strep/pcr.htm). Each serotype gives rise to one DNA fragment in only one of the PCR reactions. The size of the PCR fragment specifies the *cps* clusters and the serotype has to be confirmed by DNA sequence analysis.

**Figure 1:**
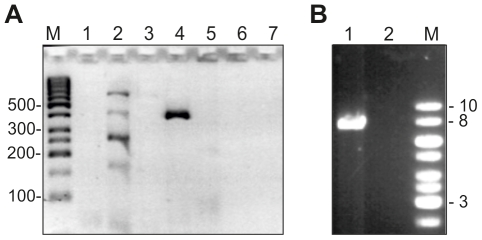
PCR products obtained from the Taï chimpanzee (Loukoum) sample. A. Multiplex PCR. Seven PCR reactions were performed as described in Materials and Methods (lanes 1–7). M: Marker DNA (GeneRuler 50 bp DNA Ladder; Fermentas). Lane 4 shows the 371 bp PCR fragment of *cps*3_Taï_. B. Amplification of the *cps*_Taï_ locus. With the primers dexB-for and aliA-rev, a long-range PCR reaction was performed. 1: Taï sample; 2: negative control (no DNA); M: GeneRuler 1 kb DNA Ladder (Fermentas).

An appr. 0.4 kb DNA fragment was obtained with all Taï samples in one of the multiplex reactions (for example, see lane 4 in Fig. 1A). However, no product corresponding to the expected *cpsA* fragment was detected in any of the PCR reactions, suggesting some unusual composition of the *cps*_Taï_ cluster. One PCR reaction resulted in several DNA fragments which did not correspond to any of the potential products, and these were not investigated further (lane 2 in Fig. 1A). DNA sequencing identified the same 371 nucleotide (nt) sequence in all three Taï samples corresponding to a *galU* fragment typical for the *S. pneumoniae cps*3 cluster (also named *cps3U* or *cap3C* [26], [27]). In this context it should be pointed out that the nomenclature proposed by Bentley *et al.* for *cps* genes was used throughout the manuscript [10].

In order to understand why the control *cpsA* fragment was not obtained, and to gain more information about the genetic arrangement of the *cps*_Taï_ cluster, a long-range PCR reaction was performed to obtain the DNA sequence of the entire *cps*3_Taï_ cluster. Primers specific for the genes *dexB* (spr0310) and *aliA* (spr0327) which are flanking all *S. pneumoniae cps* clusters were used. The PCR products from all three Taï samples were approximately 8 kb long (for example, see Fig. 1B). However, the *cps*3 region of strain *S. pneumoniae* SP3- BS71, a representative of a major type 3 clone of ST180 whose genome sequence is available, is predicted to be 12.8 kb [28], and of another type 3 *S. pneumoniae* 524/62 of unknown ST is 10.3 kb [10], a variation due to the presence of highly variable transposase fragments. The smaller size of the Taï PCR product suggests either a modified *cps*3 cluster with large deletions, or the presence of a novel capsular type in the Taï samples. DNA sequence analysis of all three 8 kb fragments clearly identified the four genes specifying the *cps*3 cluster, and all samples produced identical DNA sequences. However, the *cps*3_Taï_ region bears special features as outlined below.

### DNA sequence analysis of the *cps*3_Taï_ cluster

**Figure 2:**
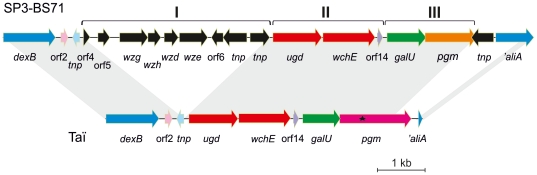
Schematic representation of the *cps*3 locus of *S. pneumoniae* SP3-BS71 compared to *cps*3_Taï_. Homologous genes are illustrated by the same colour. The *S. pneumoniae* SP3-BS71 genes (Acc. No. NZ_AAZZ01000001) missing in *cps*3_Taï_ are indicated by black arrows. Grey areas designate regions of >97% identity. ★: authentic stop codon in *pgm*_Taï_.

The *cps*3 cluster can be devided into three regions (Fig. 2) [10], [26], [29]. The first region contains sequences common to all serotypes (region I in Fig. 2), but is not required in *cps*3 since it is mutated and contains mainly pseudogenes of variable size. This entire region I is missing in *cps*3_Taï_, which explains the smaller size of the PCR product and the failure to detect the control *wzg* fragment in the multiplex PCR.

**Figure 3:**
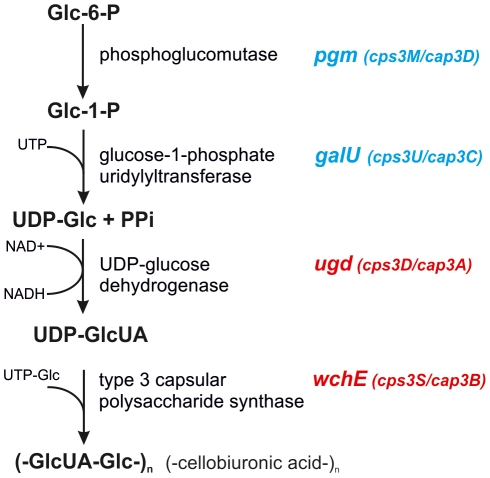
Biosynthetic pathway for type 3 capsular polysaccharide. The two genes coloured in red are specific for *cps*3. The blue colour indicates the two genes that are dispensable in the *cps*3 locus because homologues occur elsewhere in the *S. pneumoniae* genome. The nomenclature suggested by Bentley *et al.* was used [10]. Alternative gene names introduced by Dillard *et al.* [27] and Arrecubieta *et al.* [26] are indicated in brackets.

Region II contains the two genes essential for biosynthesis of the type 3 capsule which is composed of cellobiuronic acid units connected in a β(1→3) linkage [30]: *ugd* encoding the UDP-glucose dehydrogenase responsible for UDP-glucuronic acid (UDP-GlcUA) synthesis, and the type 3 synthase gene *wchE* encoding a processive β-glucosyltransferase linking the alternating glucose and GlcUA moieties (Fig. 3) [27], [29], [31]. WchE represents the simplest synthesis and export pathway for *cps*. In *cps*3_Taï_, region II is intact.

Region III contains the two genes *galU* and *pgm*. GalU and Pgm are required for synthesis of UDP-Glc (Fig. 3), a precursor for all capsular types and other cell wall polymers as well. These two genes are non essential for CPS3 biosynthesis since homologues of both genes occur elsewhere in the pneumococcal genome, here referred to as *galU*_2_ and *pgm*_2_, [27], [32]. Also, *pgm* within the *cps*3 cluster is truncated, and the putative product is probably non functional due to the lack of a C-terminal domain important for phosphomutase activity [29], [33].

**Figure 4:**
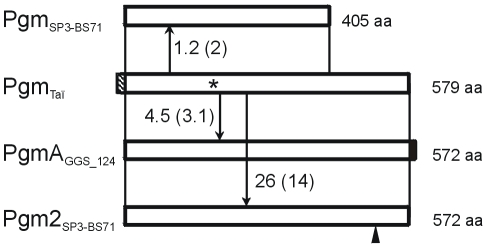
Schematic representation of Pgm_Taï_ and homologues. The numbers indicate % difference in nucleotide sequence; % differences in amino acid (aa) composition are given in brackets. The black arrow indicates an extra aa in Pgm*_2_* at position 509. Pgm_Taï_ has an eight aa N-terminal extension, and PgmA of *S. dysgalactiae* subsp. *equisimilis* GGS_124 has one extra C-terminal amino acid. The sequences from the following strains were used (Acc.No.): *S. pneumoniae* SP3_BS71 (Acc. No. NZ_AAZZ01000001); *pgmA*: *S. dysgalactiae* subsp. *equisimilis* GGS_124 (BAH81542.1). *pgm_2_*: SP3_BS71 (ZP_01818094).

Region III of *cps*3_Taï_ contains downstream of *galU* a *pgm* homologue which has some peculiar properties. The DNA sequence of *pgm*_Taï_ reveals a full size gene (1740 nt) similar in length to *pgm*_2_ (1719 nt) in contrast to e.g. *pgm*_SP3- BS71_ (1218 nt). A mutation within the ATG*_pgm_* start codon in combination with a single nucleotide deletion four nucleotides upstream results in an 8 amino acid (aa) extended N-terminal sequence of the putative *pgm*_Taï_ gene product; these mutations also affect *galU* so that it lacks the last codon. Moreover, the *pgm* codon 179 is changed into a premature stop codon resulting in a pseudogene. The DNA-sequence of the 5′-region of *pgm*_Taï_ is highly related to *pgm*_SP3-BS71_ (1.2% difference), but largely different to *pgm*_2 SP3-BS71_ (26%). Interestingly, BLAST analysis revealed a homologue *pgmA* of *S. dysgalactiae* subsp. *equisimilis* of similar size (1719 nt) which differed in only 4.5% (Fig. 4 and S1; Table 1). This strongly suggests that the Pgm gene which is located in the *cps*3 cluster is an orthologue of the chromosomal gene *pgm2* of *S. pneumoniae*.

**Table 1:** Comparative sequence analysis of *cps*3 genes.

Nucleotides				
	*ugd*	*wchE*	*galU*	1 *pgm*
524/62	4 (0.3%)	3 (0.2%)	1 (0.1%)	13 (1.4%)
WU2	4 (0.3%)	15 (1.4%)	3 (0.3%)	n.a.
406	4 (0.3%)	4 (0.3%)	2 (0.2%)	7 (0.6%)
Taï	9 (0.8%)	19 (1.5%)	8 (0.9%)	15 (1.2%)

Amino acids				
	Ugd	WchE	GalU	Pgm
524/62	2 (0.5%)	2 (0.5%)	1 (0.3%)	6 (2%)
WU2	2 (0.5%)	11 (2.6%)	1 (0.3%)	n.a.
406	2 (0.5%)	2 (0.5%)	0 (0%)	6 (1.5%)
Taï	3 (0.8%)	6 (1.4%)	2 (0.7%)	8 (2%)

The number of changes compared to those of *S. pneumoniae* SP3_BS71 are given.

1 *pgm* in strain 406 terminates with codon 306.

n.a.: not available.

**Figure 5:**
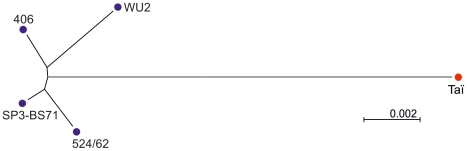
Tree showing the position of *cps*3_Taï_ within other *cps*3 clusters. A neighbour-joining radial tree was constructed as described in Materials and Methods using the region including *ugd*, *wchE* and *galU* together with the promoter region common to all samples. The optimal tree with the sum of branch length = 0.02199305 is shown. The tree is drawn to scale, with branch lengths in the same units as those of the evolutionary distances used to infer the phylogenetic tree. There were a total of 3823 positions in the final dataset.

In addition to the SP3-BS71 *cps*3 sequence [28], there are another three sequences of *S. pneumoniae cps*3 clusters available: those from strains 524/62 [10], 406 [26], and WU2 [27]. The genes of all clusters are closely related when compared among each other differing in between 1–4 nucleotides per gene except for a short highly variable region within *wchE* of the WU2 strain. However, they all differed considerably to the *cps*3_Taï_ sequence with 9 to 18 bp changes per gene (Fig. S2 and Table 1). Figure 5 shows a neighbour joining tree for the 3823 bp region including the promoter and *udg/wchE/galU*, clearly documenting that the *cps*3_Taï_ genes are more distantly related to any of the human samples than these are to each other (Fig. 5).

In the regions flanking the *cps*3 cluster differences between the ape and the human samples are also noteworthy. In the 3′-region flanking *aliA*_Taï_, a large 1.6 kb deletion has occurred (see Fig. 2). However, the AliA gene in the genome of SP3-BS71 is also affected, since the integration of a transposase resulted in deletion of a small part of the *aliA* 5′-region.

Expression of the *cps*3 genes is driven by a strong promoter upstream of *udg* [34]; and references within] and 112 bp of *cps*3_Taï_ correspond to this region. There are 12 alterations (10 substitutions, one deletion A_−48_ and one insertion T_−23_ with A_1_TG representing the start codon of *udg*). However, they do not include a mutation described within the −35 region that affects expression considerably [34], and the +1 position as well as −10 and −35 regions are well conserved in *cps*3_Taï_ sample. It is therefore likely that all genes in the *cps*3_Taï_ cluster can be expressed.

### Transformation of the unencapsulated *S. pneumoniae* R6 gene with *cps*3_Taï_

**Figure 6:**
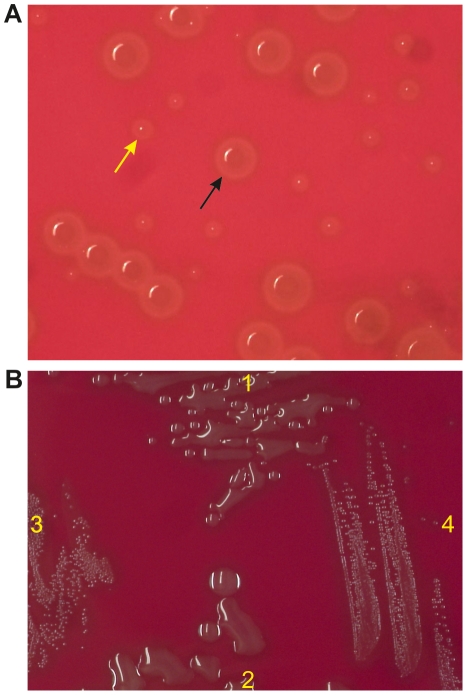
Colony morphology of *S. pneumoniae* type 3 strains. A. *S. pneumoniae* R6 transformants. After transformation with the ligation mixture (Taï-DNA and pSW1) cells were plated on trimethoprim containing D-agar. Black arrows: mucoid capsular colonies; yellow arrows: non capsulated colonies carrying religated vector. B. Serotype type 3 strains. 1: R6*bgaA*::*udg-wchE* contains the *cps*3 locus of the Taï sample; 2: SP3-BS71 represents a type 3 human isolate. 3: the parental strain R6 and 4: R6*bgaA*::pSW1 carrying only the vector pSW1 are shown as unencapsulated control strains. Pictures were taken using a Sony DFW-X700 camera.

In order to see whether the genes of the *cps*3_Taï_ cluster can be expressed from its promoter, and whether they indeed encode functional products, a 3 kb PCR fragment including the promoter region plus *ugd* and *wchE* was ligated into pSW1 as described in the Materials and Methods section. The ligation mixture was then used to transform the unencapsulated laboratory strain R6 which contains a deletion in its *cps*2 cluster [35]. Since *S. pneumoniae* R6 contains *pgm_2_* and *galU_2_* corresponding to spr1351 and spr1903, respectively, their functions were expected to complement the enzymatic machinery required for CPS3 synthesis. The ligation mixture was used as donor DNA, since wildtype colonies resulting from transformation with the religated vector fragment should easily be distinguishable from transformants containing the vector plus the 3 kb fragment and thus expressing a polysaccharide capsule. Trimethoprim resistant colonies were obtained readily, and indeed two types of colonies were apparent: appr. 40% showed no difference to the small colonies of the parental strain R6, whereas 60% had a striking mucoid phenotype typical for the type 3 capsule (Fig. 6A). This phenotype was stably maintained during several passages of single colonies (Fig. 6B). The presence of capsular material of type 3 was further verified using type 3 antiserum in a Quellung reaction (not shown). Integration of the 3 kb fragment into the *bgaA* locus was confirmed in six mucoid colonies by PCR using primers flanking the integration site. Thus, *ugd*_Taï_ and *wchE*_Taï_ in combination with *pgm_2-R6_* and *galU_2-R6_* were sufficient to drive biosynthesis of the capsule 3 polysaccharide.

## Discussion

The presence of the *S. pneumoniae* specific *cps*3 cluster in samples from dead wild apes confirmed the presence of pneumococci in the deceased animals. The samples investigated here represent both clones that were identified previously STs 2308 and 2309 [24], and were taken seven years apart. Although the allelic profile of the two clones is completely distinct, they contained identical DNA sequences of the *cps*3 cluster that differed largely from that of other type 3 isolates. It is also remarkable, that among the over 6300 STs listed in the MLST data base in April 2011, no human isolate has the same ST compared to that of the chimpanzee associated *S. pneumoniae* but differs in at least four out of the seven alleles used for MLST. Several distinct STs for type 3 isolates are known, with ST458 predominating in South Africa [36], whereas ST180 is the dominant clone in many other countries [37]–[40]. The unique *cps*3_Taï_ sequence adds further evidence that the two clones in the Taï National Park occur endemically, and suggests some selective advantage favouring recent acquisition of this CPS type. Serotype 3 is among the serotypes with the highest invasive capacity in human [41], and it is thus likely that *S. pneumoniae* played a substantial role in causing the death of the chimpanzees even though other pathogens have probably contributed to the disease [24].

The capsule is one of the major virulence factors of *S. pneumoniae*. Clones associated with animals held in captivity or as pets expressed many different serotypes, and most clones were identical to human isolates. However, guinea pigs seemed to be infected by a new clone of serotype 19F [17], and new clones of serotype 3 were isolated from racing horses [17], [22]. The identification of serotype 3 clones in wild animals described in the present manuscript is another example suggesting that specialized *S. pneumoniae* clones can be associated with animals. It has been suggested that the animal host of the Taï clones is not the chimpanzee but small rodents or monkeys that are part of the ape's diet [24]. The reason for the persistence of the *S. pneumoniae* clones in the Taï National Park is not clear. We do not believe that the capsule itself is involved in this property, since there is no indication that the capsule of the Taï samples is biochemically distinct from the known type 3 structure. It is more likely that other genomic components of these pneumococcal clones are responsible for their capacity to persist in this area. Also studies on the virulence potential of these clones have to await the isolation of the bacteria which has not been possible so far.

There are only four genes required for biosynthesis of CPS3 (Fig. 3). The two genes *ugd* (UDP-Glc dehydrogenase) and *wchE* (CPS3 synthase) involved in the last two steps are essential. The other two genes located in the *cps*3 locus - *pgm* catalyzing the production of Glc-1-P from Glc-6-P, and *galU* converting Glc-1-P to UDP-Glc - are dispensable, since homologues *galU_2_* and *pgm_2_* are present elsewhere in the *S. pneumoniae* genome. It is peculiar, that not only the truncated *pgm* gene within the *cps*3 cluster can be deleted without affecting CPS3 production, but that also deletion of *galU* has no effect, whereas mutants in *galU_2_* or *pgm_2_* produced almost no CPS3 and were strongly affected in virulence [32], [42]. This documents that it is the two genomic genes outside the *cps* locus that are mainly involved for CPS3 biosynthesis rather than their homologues in the *cps*3 cluster. The fact that transformation of the *ugd-wchE*_Taï_ region into the unencapsulated *S. pneumoniae* R6 strain results in type 3 colonies as shown here documents that the absence of both genes *galU* and *pgm* simultaneously in the *cps* locus has no apparent impact on CPS3 production and thus clearly defines the minimal size of the *cps*3 region required for CPS3 synthesis. It also proves that the *cps*3_Taï_ cluster is functional despite considerable alterations in the promoter region as well as in *udg* and *wchE*.

The comparative DNA sequence analysis of *cps*3_Taï_ revealed several features that document an evolutionary history distinct from all other known *cps*3 loci. RFLP analysis of restriction digests from WU2 and another four type 3 strains confirmed a high degree of uniformity of this locus including the transposon upstream of the AliA gene flanking the *cps* cluster [29]. However, *cps*3_Taï_ is at least 2 kb shorter due to the absence of region I (see Fig. 2), and a 3′-region that includes a transposon as well as substantial parts of *aliA* is also missing. The AliA gene is generally truncated in *cps*3 clusters [29]. Probably *aliA* is not required in *S. pneumoniae* due to the presence of several other related oligopeptide permease genes [43]. Nevertheless, AliA mutants have been shown to colonize the nasopharynx considerably less using the type 2 strain D39 [44], and thus other factors might compensate this defect in the serotype 3 isolates of high virulence potential.

The four *cps*3 loci where sequence information is available are more similar to each other than they are to *cps*3_Taï_ (Figs. 5, S1, S2 and Table 1). Furthermore, the Pgm_Taï_ gene is unique in that it represents a full size homologue in contrast to the truncated *pgm* versions in the other *cps*3 loci including those found among recently shot gun sequenced *S. pneumoniae* isolates (http://www.ncbi.nlm.nih.gov/sutils/genom_table.cgi), and again the Pgm_Taï_ gene is more different compared to all others (Fig. 4). Remarkably, the G+C content of *pgm* resembles that of *S. pneumoniae* genomes and other streptococci with 41.3%, whereas the G+C of other *cps*3 genes is significantly lower (34–37%), similar to CPS synthesizing genes in other *cps* loci [10]. In summary, two conclusions can be drawn from these data. The *cps*3 cluster contains genes from at least two sources as judged from the G+C content. Furthermore, *cps*3_Taï_ has evolved separately for a certain time period before diversification of the other *cps*3 clusters has occurred, resulting in a higher percentage of mutations and distinct deletion events. The availability of sequences from other type 3 *S. pneumoniae* clones would be desirable to broaden our understanding on the evolution of this cluster.

## Methods

### Bacterial strains and media

*S. pneumoniae* R6, a nonencapsulated derivative of the Rockefeller University strain R36A [45], was used for transformation experiments. Cells were grown at 37°C without aeration in C-medium [46] supplemented with 0.2% yeast extract (Difco) or on blood agar plates (D-agar supplemented with 3% defibrinated sheep blood (Oxoid) [47]. Growth in liquid culture was monitored by nephelometry.

*Escherichia coli* strain DH5α was used for propagation of plasmid pSW1. *E. coli* strains were grown aerobically at 37°C either in LB medium or on LB agar plates [48]. Plasmid pSW1 was selected in *E. coli* with 200 µg/ml ampicillin.

### Transformation procedure

Transformation of *S. pneumoniae* R6 strains was performed according to published procedures [49]. Transformants containing pSW1 were selected with trimethoprim at 15 µg/ml.

### DNA manipulations

All DNA techniques were performed using standard methods [48]. Multiplex PCR for 39 capsular serotypes/serogroups was performed by using seven sequential reactions as described by Pai *et al.* [25]. The primer sets specific for Africa clinical specimen were used as described by the CDC (http://www.cdc.gov/ncidod/biotech/strep/pcr.htm). PCR reactions were performed using GoldStar *Taq* polymerase (Eurogentec) according to the manufacturer's instructions. DNA isolated from three deceased chimpanzee lung tissue samples was used: Loukoum (1999, North community, ST2308), Candy (2006, East community, ST2308) and Ophelia (2004, South community, ST2309) [24]. The *cps* cluster was amplified using primers located in the genes *dexB* (dexB-for CATCATGGACTTGGTGGTCAATCATACCTCGGATGAG) and *aliA* (aliA-rev TAGACAAGATTGGACGCCCTGTACGAGATGTAGTTGG). Long-range PCR were performed using high-fidelity iProof polymerase (Bio-Rad) according to the manufacturer's instructions. The amplified products were sequenced by primer walking.

PCR products were purified using the PCR clean-up gel extraction kit (Macherey-Nagel). Chromosomal DNA was isolated from *S. pneumoniae* as described previously [50]. Plasmids from *E. coli* were isolated using the QIAprep Spin Miniprep kit (Qiagen). Restriction nucleases and T4 DNA ligase were purchased from BioLabs and used according to the recommendations of the suppliers.

### Construction of R6bgaA::*udg-wchE*

The region covering promoter and the two genes *udg* and *wchE* essential for type 3 capsular polysaccharide biosynthesis was PCR amplified using oligonucleotides pDD01 (CGCGGATCCACCGATAGTGTGGTTAATGTTG) and pDD02 (CTAGCTAGCCCAGCCCTGCTGCAGGAATAACAG), treated with *Bam*HI and *Nhe*I, and ligated to pSW1 previously digested with the same enzymes. The ligation mixture was used to transform *S. pneumoniae* R6, and trimethoprim resistance colonies were selected. Approximately 60% of the transformants displayed mucoid colony appearance and correct integration of the insert into the genome was confirmed by PCR.

pSW1 plasmid contains a pBR322-derived origin of replication for replication in *E. coli* but not in *S. pneumoniae*, and details will be described elsewhere. Briefly, it carries a trimethoprim resistance marker [51] which can be used for selection of the transformants in *S. pneumoniae*, and the β-lactamase gene (*bla*) confers ampicillin resistance in *E. coli*. Genes of interest can be cloned via multiple cloning sites with recognition sequences for *Kpn*I, *Sma*I, *Xba*I, *Bam*HI, *Sal*I. Flanking regions are homologous to *S. pneumoniae* sequences allowing integration into the chromosome by double crossover at the *bgaA* locus thereby replacing an intergenic region between *bgaA* and the adjacent gene spr0566.

#### Quellung reaction

The strains were serotyped by Quellung reaction using type serum 3 provided by the Statens Serum Institut, Copenhagen, Denmark [52].

#### Phylogenetic analysis

The evolutionary history of *cps* genes was inferred using the Neighbour-Joining method [53]. Evolutionary distances were computed using the Maximum Composite Likelihood method [54]. All positions containing gaps and missing data were eliminated from the dataset (complete deletion option). Phylogenetic analyses were conducted in MEGA4 [55].

#### Nucleotide sequence accession number

The DNA sequence described here (*cps*3_Taï_) is deposited in GenBank under accession No. JF836868.

## Supporting Information

Figure S1 **Comparative sequence analysis of the Pgm_Taï_ gene.** Shown are sites where at least one sequence differs from the reference sequence. A: nucleotide sequence; B: amino acid sequence. The codon numbers (amino acids) are indicated vertically in the first three rows; sites 1, 2 and 3 refer to the first, second and third positions in the respective codon. The authentic stop codon in *pgm*_Taï_ occurs at codon 179 and is indicated by (*) in the amino acid alignment. The sequences from the following strains were used (Acc.No.): *S. pneumoniae* 524/62 (CR931634); 406 (Z47210), SP3_BS71 (Acc. No. NZ_AAZZ01000001); *pgmA*: *S. dysgalactiae* subsp. *equisimilis* GGS_124 (BAH81542.1).
(TIF)

Figure S2 **Comparative sequence analysis of** ***cps*** **3_Taï_ genes and deduced proteins.** Shown are sites where at least one sequence differed from the reference sequence. Top: amino acid sequence; bottom: nucleotide sequence. The codons (amino acids) as indicated vertically in the first three rows are numbered according to published sequences; sites 1, 2 and 3 refer to the first, second and third positions in the respective codon. A region in *wchE* highly divergent in *S. pneumoniae* WU2 between codon 223.2 until 235.3 is shaded in grey; it includes 12 nt substitutions and 3 nt deletions resulting in two frameshifts within this region and the deletion of one amino acid in the deduced gene product. Sequences: WU2 (Acc. No. SPU15171); other Acc. Nos.: see legend to Fig. S1.
(TIF)
